# A Review of the Safety of Interleukin-17A Inhibitor Secukinumab

**DOI:** 10.3390/ph15111365

**Published:** 2022-11-07

**Authors:** Vishnu Eshwar, Ashwin Kamath, Rajeshwari Shastry, Ashok K. Shenoy, Priyanka Kamath

**Affiliations:** Department of Pharmacology, Kasturba Medical College, Mangalore, Manipal Academy of Higher Education, Manipal, India

**Keywords:** secukinumab, interleukin 17, adverse event, psoriasis, inflammatory bowel disease

## Abstract

Secukinumab is an anti-interleukin (IL)-17A IgG1-κ monoclonal antibody approved for psoriasis, psoriatic arthritis, and ankylosing spondylitis. Its efficacy is well documented, but the complete safety profile of secukinumab, especially on long-term use, needs to be studied. IL-17 inhibitors increase the risk of infections, especially respiratory tract infections and candidiasis, and inflammatory bowel disease; the causal relationships are well described. However, evidence regarding the other adverse events is scarce, and causal associations between the adverse events and the biologic remain unresolved. This review aims to present a narrative perspective on the safety of secukinumab and identify some key areas where the safety of secukinumab may potentially be useful in understanding the scope of secukinumab therapy and making informed clinical decisions.

## 1. Introduction

**Figure 1:**
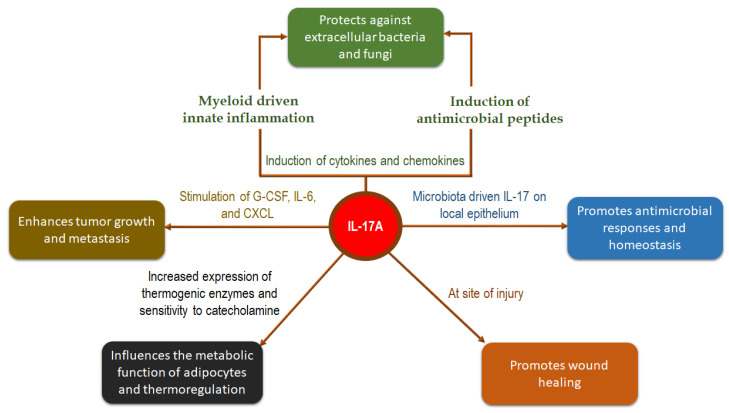
Physiological and immunological functions of IL-17A [6,7].

Secukinumab is a fully humanised immunoglobulin G1 (IgG1) κ monoclonal antibody that directly inhibits interleukin-17A (IL-17A). IL-17A is a proinflammatory cytokine; IL-17B to IL-17F are the other isoforms [1]. IL-17A and IL-17F are currently drug targets for the treatment of autoimmune diseases [2]. In normal conditions, IL-17 is involved in mucocutaneous defence [3] and immunity against extracellular pathogens [4]. However, elevated levels of IL-17 are associated with autoimmune diseases, immunopathological conditions, and cancer progression [5]. Figure 1 illustrates the physiological roles of IL-17A.

The emergence of biologics that inhibit IL-17 has provided new avenues in the maintenance therapy of many autoimmune diseases [8], in which secukinumab has proven efficacy in psoriasis, psoriatic arthritis, and ankylosing spondylitis. Though the treatment options for autoimmune diseases have increased recently, there is still little evidence in the form of efficacy studies comparing one biologic to another to make informed treatment decisions. As a result, the priority of choosing one cytokine inhibitor over another is still unclear. Secukinumab is indicated in moderate to severe plaque psoriasis and other psoriasis types. It can be started in patients who are naïve to biologics as well as in patients in whom other biologics have been ineffective or unsafe [9]. Other biologics used in psoriasis treatment are tumour necrosis factor (TNF)-α inhibitors, IL-12/23 inhibitors, and other IL-17 inhibitors. Secukinumab is also employed similarly in psoriatic arthritis, a heterogeneous inflammatory condition having musculoskeletal features associated with psoriasis [10]. In patients with ankylosing spondylitis, secukinumab is indicated when the symptoms remain unresolved with the use of nonsteroidal anti-inflammatory drugs. However, TNF-α inhibitors are preferred over IL-17 inhibitors in ankylosing spondylitis because of the availability of long-term efficacy and safety data to support the use of the former [11].

The involvement of IL-17 has also been described in certain nonpsoriatic dermatological conditions, such as hidradenitis suppurativa, pityriasis rubra pilaris, and Behçet’s disease, and secukinumab is used off-label in these conditions [12]. Secukinumab was shown to be efficacious, with a slow onset of action, in eight patients with refractory spontaneous chronic urticaria and reduced the severity and frequency of angioedema [13]. Secukinumab has also demonstrated significant improvement in papulopustular rosacea in an open-label study [14]. A child with ABCA12 deficiency-related ichthyosis showed improvement with secukinumab when given over 6 months [15]. As evidence regarding the role of IL-17 is beginning to unravel in various conditions, it becomes important to study the adverse events associated with the biologics that inhibit this cytokine. IL-17 inhibitors are novel drugs, with secukinumab being the oldest. There is little evidence of the long-term safety of this biologic.

Secukinumab has shown a favourable safety profile in clinical trials, with the most common adverse effects being upper respiratory tract infection, headache, nasopharyngitis, candida infection, hypersensitivity reaction, arthralgia, hypertension, diarrhoea, back pain, pruritus, and cough [16]. Other adverse events of interest associated with secukinumab use are neutropenia, malignant or unspecified tumours, inflammatory bowel disease (IBD), and major adverse cardiovascular events (MACE) [17]. Clinical trial data alone may be insufficient in describing the true adverse event profile of a drug used in chronic conditions. Hence, there is a need for robust pharmacovigilance measures in verifying the safety profile of the drug along with finding any new adverse event signals that are unknown yet [18].

We present here a narrative review of the safety of secukinumab, starting with the role of IL-17 in chronic immune disorders and general concerns associated with the use of IL-17 inhibitors, and then describing the literature for secukinumab and enumerating some of the important safety issues identified on the basis of available literature. The safety of secukinumab based on clinical trial data has been reviewed elsewhere [19]; we mainly focused on case reports and real-world studies in this review, with the key findings from clinical trials being mentioned briefly. The literature search methodology has been briefly described in Supplementary Material S1.

## 2. Role of IL-17 in Psoriasis and Related Disorders

Psoriasis is a chronic immune condition of the skin characterised by hyperproliferation and keratinocyte activation, manifesting as grey, scaly, erythematous plaques/lesions on the skin [20]. The role of many proinflammatory cytokines, such as TNF-α, IL-12, IL-17, IL-23, and interferon-γ (INF-γ), has been established [21]. The secretion of IL-17 is achieved through a number of immune cells, such as macrophages, dendritic cells, natural killer cells [22], and T-cells, driven with the help of T-helper cell 17 (Th17) [6]. IL-17 drives inflammation by increasing the levels of the psoriatic autoantigen, antimicrobial peptide LL37, which in turn increases the levels of another psoriatic autoantigen, ADAMTS-like protein, which then increases the expression of IL-17 and INF-γ, forming a positive feedback loop [23]. In addition, IL-17 also promotes the production of proinflammatory cytokines such as IL-6, IL-8, granulocyte colony-stimulating factor, granulocyte-macrophage-colony-stimulating factor, and chemokine (C-C motif) ligand 20, further driving inflammation [24]. IL-17 also induces keratinocyte differentiation by forming a receptor complex through binding to the IL-17 receptor; the complex then binds to epidermal growth factor-α, which induces various signal transduction pathways, ultimately increasing keratinocyte levels [25]. The inflammatory phenomenon, coupled with the role of IL-17 role in influencing the proliferation of keratinocytes, is seen to be a key factor for the manifestation of psoriatic plaques. Psoriatic arthritis is a heterogenous immune condition having the features of both psoriasis and inflammatory arthritis [26]. IL-17 has similar effects in the synovial fluid, prolonging inflammation and inducing synovitis [27].

Ankylosing spondylitis is an autoimmune condition that affects the spine joints, causing severe long-term pain [28]. It is characterised by damage to the sacroiliac joints and spinal ankylosis due to new bone formation [29]. IL-17 promotes bone growth and regeneration by inducing the proliferation and differentiation of osteoclasts [30], along with inflammation which may worsen the radiographic progression of the disease [31].

## 3. Safety Concerns with IL-17 Inhibitors

Biologics are genetically engineered drugs [32] of biomolecular origin, i.e., proteins, nucleic acids, sugars, or a complex combination of these substances [33]. Monoclonal antibodies are generally well tolerated; however, serious, rare, and unpredictable adverse drug reactions are associated with their use [34]. Unlike chemically synthesised drugs, the adverse drug events that occur as a consequence of monoclonal antibody therapy are target-related and associated with the biological consequences of their action [35]. As far as IL-17 inhibitors are concerned, there are three different mechanisms by which the inflammatory signalling is inhibited: secukinumab and ixekizumab inhibit IL-17A [36,37]; bimekizumab inhibits IL-7A and -17F [38]; brodalumab inhibits IL-17RA and -17RC receptors [39].

Since there are considerable differences in the mechanism of IL-17 inhibition, there may be variations in the adverse events exhibited by individual monoclonal antibodies. A clinical trial comparing the safety and efficacy of bimekizumab versus secukinumab in patients with plaque psoriasis showed higher rates of oral candidiasis in those receiving the former drug, although the overall rates of adverse events, including serious adverse events, were similar in both groups [40]; this may be due to the dual-target inhibition of IL-17A and IL-17F. Inhibition of IL-17RA and IL-17RC hinders the actions of not just IL-17A but also its isoforms, which may also produce variations in the adverse event profiles of IL-17 inhibitors. Similarly, brodalumab is contraindicated in Crohn’s disease [41], whereas ixekizumab and secukinumab have warning labels in the prescribing information [42,43]. Hence, there is a need for more evidence to understand the safety profile of each monoclonal antibody in IL-17 inhibition. Pichler proposed a classification of adverse events on the basis of the use of monoclonal antibodies, which can aid in identifying and classifying newly discovered adverse events [44], such as reactions caused by high levels of cytokines, hypersensitivity reactions, those due to immune or cytokine imbalance, symptoms caused by cross-reactivity, and nonimmunological reactions. Table 1 lists the important adverse effects of secukinumab according to Pichler’s classification.

**Figure 2:**
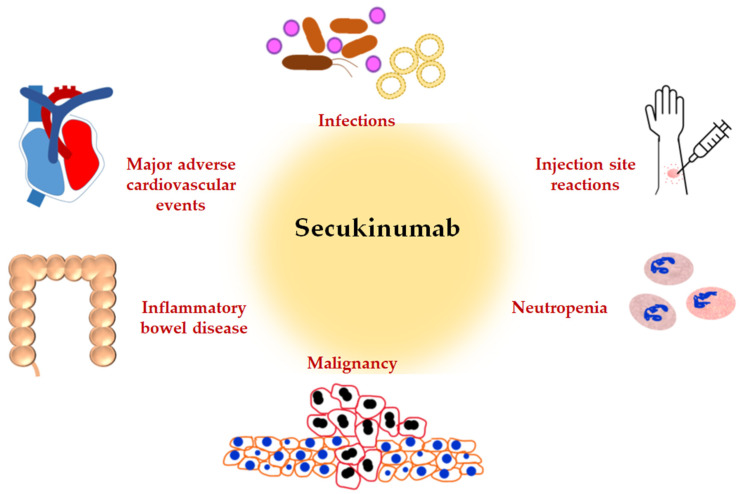
Potential adverse effects of secukinumab.

Many proinflammatory cytokines are associated with the progression of autoimmune diseases. Monoclonal antibodies selectively suppress these cytokines leading to alteration in immune homeostasis and physiological responses. This may directly affect the incidence of adverse events that are unique to each target cytokine. A meta-analysis of short-term efficacy and safety of biologicals for moderate to severe plaque psoriasis found secukinumab to have the second-highest risk of adverse events following ixekizumab [45]. Three cases of tooth abscess were reported in a pharmacovigilance study conducted in Italy assessing the safety of biologics in rheumatology [46]. Another pharmacovigilance study assessing the safety of biologics in psoriasis did not identify any adverse drug events with secukinumab [47]. In addition, a study aimed to understand the use of secukinumab in Asian and Middle-Eastern populations did not identify any new adverse signals [48]. In a real-world study involving Japanese patients, the most commonly reported adverse reaction was oral candidiasis (2.9%); the incidence of IBD was low, two patients developed tuberculosis, and the percentage of patients who developed cardiac adverse events was 2.3% out of 306 patients [49]. The important adverse effects of secukinumab are depicted in Figure 2 and described in detail below.

## 4. Important Adverse Effects of Secukinumab

### 4.1. Infections

Cytokine inhibition leads to a diminished inflammatory response, especially adaptive immunity, against pathogens. IL-17 is involved in host immunity against extracellular bacteria and fungi, which may explain the higher incidence of infections and candidiasis [50]. Autoimmune diseases themselves are a risk factor for infection [51]. Upper respiratory tract infections are common with the use of monoclonal antibodies, including secukinumab use [17]. A register-linked cohort study of Swedish patients with psoriasis showed the occurrence of respiratory and urinary tract infections to be slightly higher with secukinumab than with ustekinumab with a hazard ratio of 1.22 (95% CI: 1.03–1.43) [52]. A systematic review and meta-analysis suggested that the most occurring immune system adverse events in patients with ankylosing spondylitis treated with IL-17 inhibitors were mucosal and cutaneous infections [53].

Opportunistic infections, including tuberculosis, herpes zoster, pneumocystis jiroveci, legionella, and histoplasmosis, pose a safety concern with the use of monoclonal antibodies. Management of the opportunistic agents includes screening, immunisation, chemoprophylaxis, or treatment with antimicrobial agents [54,55]. A pooled cohort analysis of clinical trial data revealed no cases of active tuberculosis, and 0.1% of patients developed latent tuberculosis [56]. Herpes zoster infection occurred in 12/221 patients with an exposure-adjusted incidence rate (EAIR) of 2.9 per 100 patient-year [57]. A solitary case of viral pericarditis was seen in a 2-year observational study on 43 patients with palmoplantar psoriasis [58]. Two cases of cellulitis were observed in an observational study of 63 patients, where one patient had preseptal cellulitis and required hospitalisation, and the other discontinued treatment [59]. Another patient in the same study developed pneumonia requiring intensive care; secukinumab was discontinued in this patient [59].

Herpes simplex keratitis has also been described with the use of secukinumab [60]. A patient with psoriasis and psoriatic arthritis with hepatitis B was well-maintained on secukinumab with a follow-up of 2 years [61]. Similar results were also expressed in a case series of four patients with hepatitis B, although the authors have shared their concerns over the disparity of patient management at the individual level [62]. Long-term studies are required to establish the safety of secukinumab in patients with the hepatitis B virus. The occurrence of histoplasma capsulatum infection in a 45-year-old man with ankylosing spondylitis on secukinumab therapy has also been described [63]. Case reports of infections with secukinumab use are highlighted in Table 2.

### 4.2. Candidiasis

Candida infection is the most common opportunistic fungal infection observed in immunocompromised patients. Candida species in the gut are hypothesised to worsen psoriasis by stimulating nonspecific T cells and superantigens contributing further to the inflammatory cascade observed in psoriasis [68]. IL-17 is involved in neutrophil recruitment, the release of antimicrobial peptides, and the protection of mucocutaneous barriers [69]. Impairment of this function by IL-17 inhibitors used in psoriasis is causal with a candida infection. Both oral and gastrointestinal candidiasis manifestations are observed with secukinumab use. A pooled analysis of clinical trial data revealed that all cases of candidiasis were mild to moderate in severity; no cases of systemic candidiasis were reported; the EAIR were 2.2, 1.5, and 0.7 per 100 patient-years in psoriasis, psoriatic arthritis, and ankylosing spondylitis groups, respectively [70]. A postmarketing study revealed that the incidence of candidiasis is 4–10 times higher in patients treated with IL-17 inhibitors compared with those treated with TNF-α inhibitors [71]. Such cases can be managed using clotrimazole troche, nystatin suspension, miconazole mucoadhesive buccal tablet, or oral fluconazole for 7–14 days [72]. Case reports of candidiasis are described in Table 3.

### 4.3. Injection Site Reactions

Monoclonal antibodies are proteins susceptible to gastrointestinal degradation. As a result, they are administered parenterally to attain clinically relevant plasma concentrations. The preferred route of administration of biologics is the subcutaneous route. Self-injector devices have made the administration of monoclonal antibodies easier. However, injection site reactions (ISR), such as swelling, erythema, pruritus, and pain around the site of injection [79], are seen with the use of monoclonal antibodies and biologics in general. ISR occurs either due to the excipients or the drug itself and can be irritative or allergic reactions [80]. A phase I study assessing the pharmacokinetics and tolerability of subcutaneous formulations of secukinumab injected using different devices showed the occurrence of erythema, induration, haemorrhage, pruritis, and leakage. However, apart from erythema, the overall incidence of the other ISR is low [81]. In contrast, a postmarketing study revealed pain, bruising, and haemorrhage to be common ISR with secukinumab use [82]; however, the incidence of erythema, pruritis, reaction (injection-site related), and swelling were higher with ixekizumab compared with secukinumab [82]. Some of the patient factors that indicate a higher risk of ISR are female gender, low body weight, and the presence of fibromyalgia, depression, or severe rheumatoid arthritis [83].

### 4.4. Neutropenia

IL-17 has a role in neutrophil recruitment, function, and survival [84]. A proposed mechanism is by inducing chemokines CXCL1 and CXCL2 [85]. Neutropenia is a prevalent complication in immunocompromised patients with significant morbidity and mortality rate [86]. Grade-3 neutropenia (absolute neutrophil count ≥500 to 1000 cells/mm^3^) and grade-4 neutropenia (absolute neutrophil count <500 cells/mm^3^) have occurred in the clinical trial setting, with the latter being rare. However, the incidence of neutropenia with secukinumab is low, with an EAIR of 0.3, 0.2, and 0.5 per 100 patient-years in patients with psoriasis, psoriatic arthritis, and ankylosing spondylitis, respectively; uncomplicated viral upper respiratory tract infection was the most commonly observed adverse event co-reported with neutropenia [70]. A retrospective study that followed up 36 patients for 6 months on secukinumab revealed no significant difference in the haematologic parameters from baseline till the end of the study [87]. This may imply that IL-17 by itself does not have a major role in the recruitment of neutrophils [88].

### 4.5. Malignancy

The association between autoimmunity and cancer is a topic of great interest. Immune cells and cytokines dysregulated in autoimmune conditions may play a role in the development of cancer [89]. A meta-analysis showed that patients with psoriasis are 1.18 times at risk of developing cancer, with a 1.22-fold increase in cancer mortality compared with psoriasis-free patients [90]; however, none of the studies in the meta-analysis adequately adjusted for treatment exposure. Another study showed that the risk of developing high-grade cervical dysplasia and cervical cancer was 1.49 per 1000 patients [91]. In addition, the risk of keratinocyte cancer, lymphomas, lung cancer, bladder cancer, lymphoma, and non-Hodgkin’s lymphoma is increased in patients having psoriasis [92]. A higher risk of lymphohematologic malignancies and lymphoma was seen in a meta-analysis of observational cohort studies [93]. In a large-scale cohort study, males with ankylosing spondylitis had a higher risk of bone, prostate, and haematological malignancies, whereas females were at an increased risk of colon and haematological malignancies [94].

IL-17 has a controversial role in tumour immunity as it is hypothesised to play a role in both tumour suppression as well as proliferation [5]. Pooled data from clinical trials and postmarketing studies showed the low and infrequent incidence of malignancy in the secukinumab-treated patient population over a 5-year follow-up period [95]. As far as warnings in prescribing information are concerned, only ustekinumab and TNF-α have warnings against malignancy (especially lymphoma). This may be due to the availability of long-term data for these monoclonal antibodies. There is a need for long-term studies to evaluate the safety of secukinumab and other IL-17 inhibitors.

### 4.6. IBD

IBD is a chronic autoimmune condition observed in the large intestine, manifesting as Crohn’s disease (CD) or ulcerative colitis (UC). IBD is well-documented comorbidity in psoriasis [96]. Patients with psoriasis are three times more likely to develop CD [97]. Earlier, it was postulated that high levels of IL-17 exacerbate IBD [85]. However, a phase II trial of secukinumab in IBD patients was terminated due to worsening disease and unsatisfactory efficacy. In this study, 4 out of 7 drug-related adverse events were worsening of Crohn’s disease, and two additional adverse events occurred: pilonidal cyst and ileostomy, which were related to the worsening of CD with secukinumab treatment [98]. The drug label of secukinumab contains warnings and precautions while administrating the drug to patients suffering from IBD. A similar incident occurred in 1999 when a TNF-α inhibitor lenercept was tried on patients with multiple sclerosis [99]. In a randomised phase II clinical trial of brodalumab, the worsening of CD represented 25% of the total adverse events, and brodalumab is contraindicated in CD [88]. Though the incidence of CD in the secukinumab trial was 15.4%, its incidence rate is similar to the placebo, whereas, with brodalumab, only 6% of the participants in the placebo group reported worsening of CD [98,100]. The increased severity found in brodalumab may be due to the inhibition of all the ligands of IL-17 through IL-17RA and IL-17 RC receptor inhibition. In contrast, secukinumab targets IL-17A with high specificity. In clinical trials, the EAIR of IBD was low; 0.01, 0.05, and 0.1 per 100 patient-year in those with psoriasis, psoriatic arthritis, and ankylosing spondylitis, respectively; this led to the discontinuation of secukinumab in all the affected cases [70].

The link between IBD and secukinumab has been identified in the postmarketing setting as well. A retrospective cohort study consisting of patients on secukinumab for ankylosing spondylitis and psoriatic arthritis found associations between secukinumab and low rates of absolute gastrointestinal-related adverse events. In addition, exacerbation of existing conditions is more likely and tends to occur within one year [101]. A retrospective analysis of Vigibase data showed anti-IL-17 use associated with an exacerbation or new onset of IBD and colitis [102]. A signal between secukinumab and IBD was also identified in the FAERS database [103]. There is no clear-cut guideline explaining the management of IBD with IL-17 inhibitor use. Based on the data from case reports, the following approaches have been used: discontinuation of the drug; treating IBD with conventional immunosuppressants [104] and/or glucocorticoids; switching biologic to either TNF-α inhibitors [105], ustekinumab [106] or tildrakizumab [107] and in one case subtotal ileectomy was performed [108]. The case reports of IBD are presented in Table 4.

### 4.7. MACE

Cardiovascular comorbidities have been linked to autoimmune diseases. Psoriasis is identified as an independent factor for myocardial infarction (MI), especially in the younger population [114]. The chance of being exposed to cardiovascular outcomes such as stroke, MI, and coronary artery disease (CAD) is higher in severe conditions [115]. Additionally, a meta-analysis assessing observational studies indicates that patients with ankylosing spondylitis are at a 1.41-fold risk of having CAD [116]. Psoriatic arthritis also increases the risk of clinical and subclinical cardiovascular disease, thus attributing to hastened atherosclerosis [117].

The role of biologics in directly precipitating cardiac adverse events is controversial. Infliximab at 10 mg/kg had significantly increased the risk of heart failure in a pilot study [118]; doses greater than 5 mg/kg of infliximab are contraindicated in moderate-to-severe heart failure. Etanercept did not show any significant cardiovascular harm [119]. A clinical trial on the effects of secukinumab in the aortic vascular inflammation in moderate-to-severe plaque psoriasis showed secukinumab to have a neutral effect on aortic vascular inflammation and biomarkers of cardiometabolic disease [120]. Case reports in the literature did show one case of IgA vasculitis associated with secukinumab [121] and one case of cutaneous vasculitis with gut involvement [122]. Pooled clinical trials and postmarketing studies showed a low incidence of MACE [70].

### 4.8. Other Adverse Events

A study evaluated the use of secukinumab in uveitis. Although it improved uveitis in that study [36], later on, it appeared as an adverse effect, which may be due to a paradoxical effect. In a pooled safety analysis, the incidence of uveitis was low (1.4/100 patient-years) [123], and two case reports of uveitis have been reported [124,125]. It should also be noted that uveitis itself has an increased chance of occurrence in patients with autoimmune diseases [126].

IL-17 is seen to play a pathophysiological role in the manifestation of nonpsoriatic dermatological diseases [127]; nonetheless, dermatological adverse events are observed with the use of IL-17 inhibitors. Adverse events such as eczema [128], hidradenitis suppurativa [129], psoriasiform eruptions [130], and pemphigus [131] are documented in case reports. The incidence of such adverse events can be explained as a paradoxical reaction attributed to the Th1/Th2 imbalance in the skin, where the latter is overexpressed in the Th1-suppressed state [132].

Brodalumab has a black box warning for suicidal tendencies, although studies did not identify a causal link between the two; however, depression has been identified as an adverse event [133,134]. There is also a concern about suicidal tendencies with autoimmune diseases per se. Patients with autoimmune diseases often lead a poor quality of life and face social stigma. It is seen that inflammation may be associated with depression [135], and cytokine levels in the blood may correlate with depression [136]. Additionally, high levels of IL-17 have been seen in anxious patients with rheumatoid arthritis [137]. However, in a pooled safety analysis, secukinumab posed no risk of suicidal tendency in 3430 patients followed for 52 weeks [138]. A case report of depression has been documented in the literature, which was managed well with the help of sertraline [139]. Other adverse events reported with secukinumab use are presented in Table 5.

## 5. Conclusions and Future Perspectives

Autoimmune diseases are heterogeneous and can affect multiple organ systems. Target-based treatments through the introduction of monoclonal antibodies have revolutionised the treatment of autoimmune diseases with reliable efficacy. However, with any medication, there are potential adverse effects that need to be assessed and identified. It is important to correlate biologic therapy with the comorbidities associated with autoimmune diseases.

Secukinumab is an anti-IL-17 agent used in the treatment of psoriasis, psoriatic arthritis, and ankylosing spondylitis. Since IL-17 plays an important role in protecting the mucocutaneous barrier, induction of antimicrobial peptides, and driving innate inflammation in response to invading pathogens, secukinumab is prone to increase the risk of infections, dermatological and ISR, and IBD. Most cases of infections can be treated using appropriate antimicrobials and do not necessarily require drug discontinuation. The varying dermatological adverse effects that follow secukinumab use may require discontinuation of the drug and use of glucocorticoids and/or other immunosuppressants, as appropriate. Patients experiencing IBD will likely require drug discontinuation; alternatives used include other anti-IL-17 drugs, non-IL-17 biologics, glucocorticoids, and nonbiologic immunosuppressants. Other safety signals require long-term studies to establish definite causal associations. Additional studies on vulnerable populations are also essential. Existing literature has found no new safety signals for secukinumab in pregnant and pediatric populations [181,182]. A retrospective study on 29 geriatric patients also revealed no new safety signals [183]. The effect of biologics on comorbidities needs to be investigated. Interventional studies on patients with existing comorbidities or observational study data can also aid in understanding the true safety profile of secukinumab.

Unlike in the early 2000s, the use of biologics has expanded substantially. The long-term safety of biologics is vital in making informed clinical decisions. Though treatment guidelines have mentioned switching to biologics, there are insufficient head-on studies establishing their relative safety and efficacy [9]. As monoclonal antibodies face patent expiry, new opportunities to manufacture biosimilars are opening. It is impossible to completely replicate the composition of a biologic; as a result, further introduction of variations may be observed in their efficacy and adverse event profiles [184]. In this case, it would be important for the safety profile of the reference biologic to be well established.

## Figures and Tables

**Table 1:** List of adverse drug reactions to secukinumab as per Pichler’s classification [44].

Adverse Event Class	Adverse Events
Type-α	None reported to date
Type-β	Hypersensitivity and injection site reactions
Type-γ	Inflammatory bowel disease, infections, allergic and atopic disorders, neutropenia, and paradoxical inflammatory adverse events.
Type-δ	None reported to date
Type-ε	Major adverse cardiovascular events, malignancy

Type-α, due to high levels of cytokines and cytokine release syndrome; type-β, hypersensitivity reactions; type-γ, immune or cytokine imbalance syndromes; type-δ, symptoms due to cross reactivity; type-ε, nonimmunological reactions.

**Table 2:** Case reports of infections reported with secukinumab use.

Author(s)	Adverse Drug Event	Indication	Age/Sex	Duration Since Initiation of Secukinumab	Previous History of Biologic Use	Concomitant Medication	Management	Discontinuation of Secukinumab
Sinha et al. [60]	Herpes keratitis	Psoriasis	35/M	4 weeks	No	NA	3% Acyclovir five times a day, topical moxifloxacin eye drops four times a day, along with topical lubricant eye drops, topical steroids, and emollients for psoriasis	NA
Wang [63]	Scleritis due to *Histoplasma capsulatum*	Ankylosing spondylitis	45/M	NA	NA	Intravitreal triamcinolone; topical prednisolone; oral prednisone	Left eye: oral itraconazole 200 mg twice daily and fortified topical amphotericin B 0.15% four times daily with a rapid taper of oral prednisone. Right eye: topical amphotericin for two months until the subconjunctival purulence resolved. Maintenance: 6-month course of itraconazole	NA
Martin et al. [64]	Staphylococcal toxic shock syndrome	Psoriasis	6/F	2 weeks	NA	NA	Levofloxacin and rifampin, followed by trimethoprim/sulfamethoxazole, and cefuroxime unt	Yes
Utiyama et al. [65]	Infective dermatitis	Psoriasis	71/F	2 months	No	NA	Sulfamethoxazole and trimethoprim followed by doxycycline.	Yes
Fisher et al. [66]	Necrotising fasciitis	Psoriasis	18/M	4 weeks	No	NA	Surgical debridement followed by intravenous antibiotics	No
Anderson et al. [67]	Invasive *Haemophilus influenzae*	Psoriatic arthritis	42/F	18 months	Yes	NA	Empiric gentamicin and metronidazole, which was narrowed to ceftriaxone and metronidazole	NA

NA, data not available.

**Table 3:** Case reports of candidiasis associated with secukinumab use.

Author(s)	Adverse Drug Event	Indication	Age/Sex	Duration Since Initiation of Secukinumab	Previous History of Biologic Use	Concomitant Medication	Management	Discontinuation of Secukinumab
Picciani et al. [73]	Oral candidiasis	Psoriasis	50/F	6 months	Yes	NA	Miconazole gel	Resumed at a lower dose after management
Kang et al. [74]	Oesophageal candidiasis	Psoriasis	61/M	3 weeks	NA	NA	Fluconazole 200 mg/day for seven days; switched to guselkumab after infection resolved	Yes
Faccini et al. [75]	Candidemia	Psoriatic arthritis	42/F	2 months	Yes	NA	Amphotericin B switched to anidulafungin 100 mg OD.	Yes
Farah [76]	Hyperplastic candidosis and oral lichenoid lesion	Psoriasis	52/F	NA	NA	Perindopril arginine, pantoprazole, mometasone furoate.	Oral	No
Capusan et al. [77]	Oral lichenoid reaction with candidiasis	Psoriasis	62/M	8 months	Yes	NA	Intralesional corticosteroids and itraconazole; switched to apremilast for psoriasis	Yes
Komori et al. [78]	Oral lichen planus with candidiasis	Psoriasis	74/F	5 months	Yes	NA	Amphotericin B syrup	Yes

NA, data not available.

**Table 4:** Case reports of inflammatory bowel disease associated with secukinumab use.

Author(s)	Adverse Drug Event	Indication	Age/Sex	Duration Since Initiation of Secukinumab	Previous History of Biologic Use	Concomitant Medication	Management	Discontinuation of Secukinumab
Achufusi et al. [105]	Ulcerative colitis	Psoriasis	39/M	6 months	NA	NA	Infliximab (symptomatic relief) and apremilast (for psoriasis)	Yes
Ehrlich et al. [109]	Ulcerative colitis	Ankylosing spondylitis	42/M	6 weeks	Yes	Naproxen; Methotrexate	Methylprednisolone for 1 month (unsatisfactory) followed by ixekizumab	Yes
Darch et al. [107]	Inflammatory bowel disease	Psoriasis and psoriatic arthritis	54/F	14 months	No	NSAIDs	Tildrakizumab	Yes
Lozano et al. [106]	Ileocolic Crohn’s disease	Psoriasis	19/F	2 months	No	NA	Corticosteroid and switched to ustekinumab	Yes
	Ulcerative colitis	Ankylosing spondylitis	60/M	3 weeks	No	Naproxen; sulphasalazine	Full-dose intravenous steroid treatment, mesalazine enemas, and initiation of infliximab for corticosteroid refractoriness	Yes
Obeidat et al. [104]	Ulcerative colitis	Psoriatic arthritis	41/F	9 months	NA	Venlafaxine, NSAIDs, and sulfasalazine	The patient was started on budesonide with significant improvement in her symptoms. Budesonide was eventually tapered, and the patient was started on azathioprine as a steroid-sparing agent and immunomodulator	Yes
Johnston et al. [110]	Ulcerative colitis	Ankylosing spondylitis	27/M	4 months	Yes	NA	Intravenous cortisone and switch to infliximab.	Yes
Shiga et al. [111]	Crohn’s disease/Behcet’s disease–like lesions	Psoriasis	56/M	8 weeks	No	NA	Oral prednisolone 40 mg OD	NA
Uchida et al. [112]	Ulcerative colitis	Psoriasis	41/F	4 months	Yes	NA	Mesalazine 2400 mg daily and switch to adalimumab 20 mg	Yes
Lee et al. [108]	Ulcerative colitis	Psoriasis	52/M	4 months	Yes	NA	subtotal colectomy	Yes
	Ulcerative colitis	Ankylosing spondylitis	38/M	3 weeks	Yes	NA	IV infliximab 5 mg/kg	Yes
Haidari et al. [113]	Asymptomatic Crohn’s disease	Psoriasis and psoriatic arthritis	69/M	18 months	Yes	NA	Ustekinumab for CD and switch guselkumab for psoriasis and psoriatic arthritis.	Yes

NA, data not available.

**Table 5:** Other adverse events reported with secukinumab use.

Author(s)	Adverse Drug Event	Indication	Age/Sex	Duration since Initiation of Secukinumab	Previous History of Biologic Use	Concomitant Medication	Management	Discontinuation of Secukinumab
Navarro-Triviño et al. [129]	Hidradenitis suppurativa	Psoriasis and psoriatic arthritis	58/M	NA	Yes	NA	Ustekinumab (45 mg) 16 weeks	Yes
Blackcloud et al. [140]	Bullous acral eruption	Psoriasis	44/F	~1 month	Yes	Halobetasol ointment; fluocinonide gel; tacrolimus ointment; metformin; spironolactone; norethindrone-ethinyl estradiol; albuterol	Cyclosporine 100 mg BD and corticosteroid wet wraps	Yes
Gerhard Eichhoff [141]	Pompholyx	Psoriasis	35/M	3 months	Yes	NA	Clobetasol propionate 0.05% cream	No
Clark et al. [142]	Granuloma annulare	Psoriasis	69/F	6 months	Yes	Lisinopril; metformin; pravastatin; citalopram; alprazolam	Rifampin, levofloxacin, and minocycline for six months (unsatisfactory), followed by etanercept for six weeks (event resolved)	Yes
Bonomo et al. [143]	Granuloma annulare	Psoriasis and psoriatic arthritis	60/M	2 weeks	Yes	Methotrexate; levothyroxine; omeprazole; duloxetine	Topical clobetasol propionate 0.05% cream	No
Zheutlin et al. [144]	Polychondritis	Ankylosing spondylitis	56/M	3–4 months	Yes	NA	Prednisone, methotrexate, and folate therapy	Yes
Hayashida et al. [131]	Pemphigus	Rheumatoid arthritis	41/F	3 months	Yes	Methotrexate; prednisone; paracetamol	Higher dose of methotrexate and topical steroid, and tocilizumab for rheumatoid arthritis	Yes
Sladden et al. [130]	Psoriasiform eruption	Psoriasis	61/F	3 months	Yes	NA	1% Methotrexate gel and ustekinumab	Yes
Dastoli et al. [145]	Erectile dysfunction	Psoriasis	45/M	2 months	No	NA	Infliximab	Yes
Peigottu et al. [146]	Drug eruption	Psoriasis	57/F	~3–4 weeks	Yes	NA	Topical and systemic corticosteroids	Yes
Hitaka et al. [147]	Angular cheilitis	Psoriasis	23/F	2 months (appeared 3 days after every secukinumab injection)	NA	NA	Adalimumab	Yes
Shibata et al. [148]	Drug eruption	Psoriatic arthritis	52/F	~2–3 weeks	NA	NA	Topical betamethasone butyrate propionate ointment for skin eruption	No
Thompson et al. [149]	Ulcerative lichenoid mucositis	Psoriasis	62/M	1 week	Yes	NA	0.1% Triamcinolone in orabase paste	Yes
Ramalho et al. [150]	Pituitary enlargement and panhypopituitarism	Psoriasis	66/M	3 years	NA	NA	Oral hydrocortisone 40 mg in the morning and 20 mg in the afternoon; levothyroxine 50 μg/day	Yes
Nadwi et al. [124]	Anterior uveitis	Ankylosing spondylitis	47/M	6 months	Yes	NA	Local corticosteroid eye drops for 2 months	No
Su et al. [125]	Uveitis	Psoriasis and psoriatic arthritis	45/M	3 weeks	Yes	NA	Infliximab 5 mg/kg	Yes
Lu et al. [151]	Cutaneous sarcoidosis	Psoriasis	36/M	45 days	No	NA	No treatment. Symptoms resolved on their own in 2 months.	Yes
Currado et al. [152]	Psoriasis	Ankylosing spondylitis	54/F	11 months	Yes	NA	Calcipotriol, betamethasone cream and oral NSAIDs	Yes
Mammadli et al. [153]	Thrombophlebitis	Psoriasis	48/M	1 week	Yes	NA	Treatment with Ustekinumab	Yes
Peera et al. [154]	Palmoplantar pompholyx	Psoriasis	65/F	7 weeks	NA	NA	Resolution of symptoms 4 weeks after secukinumab discontinuation.	Yes
	Palmoplantar pompholyx	Psoriasis	64/F	4 months	No	NA	Resolution of symptoms 1 month after secukinumab discontinuation and switch to ustekinumab	Yes
Wehrmann et al. [155]	Drug-induced lupus erythematosus	Psoriasis	52/F	5 months	NA	NA	Ustekinumab	Yes
Bose et al. [128]	Eczema	Psoriasis	52/F	8 months	No	NA	Cyclosporine and guselkumab	Yes
	Eczema	Psoriasis	69/F	7 weeks	NA	NA	Infliximab and apremilast	Yes
Roncada et al. [156]	Atopic dermatitis	Psoriasis	59/F	2 months	NA	NA	Cyclosporine, 5 mg/kg/dose; intravenous antibiotic therapy, and skin barrier restorative creams and topical corticosteroids	Yes
Dincses et al. [157]	Behçet’s syndrome	Ankylosing spondylitis	34/M	3 weeks	Yes	NA	10 mg/day of prednisolone and certolizumab	Yes
	Behçet’s syndrome	Ankylosing spondylitis	29/M	2 weeks	Yes	NA	Three pulses of methylprednisolone and infliximab 5 mg/kg	Yes
Zhang et al. [158]	Multiple lentigines	Psoriasis	46/M	3 months	NA	NA	NA	No
Dogra et al. [159]	Paradoxical pustular psoriasis	Psoriasis	22/M	9 months	NA	NA	Complete remission was finally attained after intravenous administration of infliximab 300 mg	Yes
Kobak [160]	Raynaud’s phenomenon	Ankylosing spondylitis	35/F	3 months	Yes	NA	Low-dose aspirin and calcium channel blockers	No
Fermon et al. [161]	Aphthous Stomatitis	Psoriatic arthritis	57/M	6 months	Yes	Clopidogrel, flecainide and oral potassium.	High dose of corticosteroids and then with adalimumab again	Yes
Giordano et al. [162]	Vitiligo	Psoriatic arthritis	42/F	1 year	No	NA	NA	No
Power et al. [163]	Crystalline corneal deposition	Ankylosing spondylitis	18/M	6 months	No	Budesonide, formoterol fumarate, salbutamol, and montelukast	Observed for 12 months.	No
Kirby et al. [164]	Multisystem sarcoidosis	Psoriatic arthritis	52/F	6 months	Yes	Long-acting beta-agonist and corticosteroid inhalers.	Prednisolone 30 mg by mouth daily, tapered down to 5 mg monthly.	Yes
Elias et al. [165]	Scleroderma	Psoriatic arthritis	46/F	19 months	NA	Hydrochlorothiazide and levothyroxine	Secukinumab was discontinued, and symptoms resolved gradually.	Yes
Petty et al. [166]	Pyoderma gangrenosum	Psoriasis	50’s/F	2 weeks	No	NA	Ustekinumab 90 mg	Yes
Oiwa et al. [167]	Facial erythema with dryness and pruritus	Psoriasis	49/M	3 weeks	NA	NA	Petrolatum	NA
Hayashi et al. [168]	Latent interstitial pneumonia	Psoriasis	66/M	10 Months	NA	NA	Oral prednisolone and subsequent intravenous high-dose methylprednisolone were administered	Yes
Kajihara et al. [169]	Interstitial pneumonia	Psoriasis	36/M	18 weeks	Yes	NA	Symptoms resolved 5 weeks after secukinumab discontinuation.	Yes
Noell et al. [170]	Flared Psoriasis	Psoriasis	53/F	Shortly after initiation	Yes	NA	Transition to infliximab after ustekinumab and corticosteroids.	Yes
Quach et al. [171]	Perianal dermatophytosis	Psoriasis	40’s/F	3 Months	NA	NA	Terbinafine 250 mg OD and butenafine cream BID for 1 month	NA
	Perianal dermatophytosis	Psoriasis	60’s/F	5 weeks	NA	NA	Oral amphotericin B for 14 days and terbinafine cream for 1 month	NA
Ho et al. [172]	Bullous pemphigoid	Psoriasis	65/F	8 days	Yes	NA	Clobetasol dipropionate	No
Cranwell et al. [173]	Pseudolymphoma	Psoriasis	56/M	3 days	NA	NA	Switch to topical therapy	Yes
Jin et al. [174]	Pyoderma gangrenosum	Psoriatic arthritis	47/F	4 months	Yes	NA	Oral cyclosporin, 2.5 mg/kg per day,	Yes
Benzaquen et al. [175]	Herpetiform aphthous ulcerations	SAPHO syndrome	35/F	5 weeks	Yes	NA	3 weeks with betamethasone mouthwash. Reduction of secukinumab dose to 150 mg.	No
	Herpetiform aphthous ulcerations	Psoriasis	37/F	4 weeks	Yes	NA	Switch to ustekinumab, and lesions resolved within 3 weeks.	Yes
Burlando et al. [176]	Atopic like dermatitis	Psoriasis	70/F	6 months	No	NA	Symptoms resolved after discontinuation and topicals and phototherapy for psoriasis.	Yes
Hoshina et al. [177]	Psoriatic eruptions	Psoriasis	43/F	4 weeks	Yes	NA	Cyclosporine 200 mg/day.	Yes
Wollina et al. [178]	Pyoderma gangrenosum	Psoriasis	33/F	12 months	No	NA	Systemic prednisolone 100 mg/day, pantoprazole, topical corticosteroids.	NA
Perkovic et al. [121]	IgA vasculitis	Ankylosing spondylitis	39/F	18 months	Yes	NA	Methotrexate reintroduced 15 mg/week,	Yes
Chelli et al. [122]	Cutaneous vasculitis with gut involvement	Psoriatic arthritis	54/F	1 month	Yes	NA	Prednisone and colchicine for symptomatic management. Methotrexate 15 mg/kg was restarted.	Yes
Shaheen et al. [179]	Terminal Ileitis	Psoriatic arthritis	39/M	2 months	NA	NA	Ciprofloxacin and metronidazole later switched to piperacillin-tazobactam and received total parenteral nutrition	Yes
Rahman et al. [180]	Autoimmune hemolytic anaemia	Psoriasis	39/M	8 weeks	NA	NA	No	No

NA, data not available.

## Data Availability

Data sharing not applicable.

## Supplementary Materials

The following supporting information can be downloaded at: https://www.mdpi.com/article/10.3390/ph15111365/s1.
