# Supplementary material for: A Review of the Safety of Interleukin-17A Inhibitor Secukinumab
Source: Pharmaceuticals (Basel). 2022 Nov 7;15(11):1365. doi: 10.3390/ph15111365 (PMC9695424; doi:10.3390/ph15111365)
Supplement: Supplementary file 1 [file pharmaceuticals-15-01365-s001.zip › pharmaceuticals-1944783-supplementary.pdf]

Literature search

For identifying the relevant literature, we searched the PubMed database using the terms ("secukinumab") AND ("adverse drug reactions" OR "adverse drug events" OR "safety" OR "tolerability" OR "real world safety" OR "case report" OR "case series" OR "post marketing study") AND ("psoriasis" OR "psoriatic arthritis" OR "ankylosing spondylitis"). The search terms were applied to all fields. The search yielded a total of 543 hits with 108 reviews (44 systematic reviews), 33 meta-analyses, 242 case reports, 53 randomised controlled trials and 12 observational studies. Of the 242 case reports, we considered only those which described adverse events following secukinumab use. Furthermore, additional studies were obtained from the reference lists of the assessed manuscripts. Other clinical studies and case reports were also referred which focused on the safety of secukinumab and other IL-17 inhibitors. In addition, the drug labels of IL-17 inhibitors were also referred to obtain any additional safety information.
